# Control of the C. albicans Cell Wall Damage Response by Transcriptional Regulator Cas5

**DOI:** 10.1371/journal.ppat.0020021

**Published:** 2006-03-17

**Authors:** Vincent M Bruno, Sergey Kalachikov, Ryan Subaran, Clarissa J Nobile, Christos Kyratsous, Aaron P Mitchell

**Affiliations:** 1 Integrated Program in Cellular, Molecular, and Biophysical Studies, Columbia University, New York, New York, United States of America; 2 Columbia Genome Center, Columbia University, New York, New York, United States of America; 3 Department of Microbiology, Columbia University, New York, New York, United States of America; 4 Biological Sciences Program, Columbia University, New York, New York, United States of America; Johns Hopkins University, United States of America

## Abstract

The fungal cell wall is vital for growth, development, and interaction of cells with their environment. The response to cell wall damage is well understood from studies in the budding yeast *Saccharomyces cerevisiae,* where numerous cell wall integrity (CWI) genes are activated by transcription factor ScRlm1. Prior evidence suggests the hypothesis that both response and regulation may be conserved in the major fungal pathogen *Candida albicans.* We have tested this hypothesis by using a new C. albicans genetic resource: we have screened mutants defective in putative transcription factor genes for sensitivity to the cell wall biosynthesis inhibitor caspofungin. We find that the zinc finger protein CaCas5, which lacks a unique ortholog in *S. cerevisiae,* governs expression of many CWI genes. CaRlm1 has a modest role in this response. The transcriptional coactivator CaAda2 is also required for expression of many CaCas5-dependent genes, as expected if CaCas5 recruits CaAda2 to activate target gene transcription. Many caspofungin-induced C. albicans genes specify endoplasmic reticulum and secretion functions. Such genes are not induced in *S. cerevisiae,* but promote its growth in caspofungin. We have used a new resource to identify a key C. albicans transcriptional regulator of CWI genes and antifungal sensitivity. Our gene expression findings indicate that both divergent and conserved response genes may have significant functional roles. Our strategy may be broadly useful for identification of pathogen-specific regulatory pathways and critical response genes.

## Synopsis

For microbial pathogens, the cell wall is critical for interaction with both host and environment. The major fungal pathogen, *Candida albicans,* has a cell wall that resembles that of the model yeast *Saccharomyces cerevisiae,* and much of what is known about C. albicans cell wall biogenesis and repair comes via extrapolation from *S. cerevisiae.* Here, Bruno and colleagues inquired directly into the mechanisms that C. albicans uses to respond to disruption of cell wall biogenesis by the antifungal drug caspofungin, using a genetic strategy newly developed for *C. albicans.* They found that the response itself has many similarities to that of *S. cerevisiae,* but the regulatory circuitry is distinct: the major C. albicans regulatory gene has no clear counterpart among S. cerevisiae genes. Their findings provide a new example of a unique C. albicans regulatory function and one that may prove useful in identifying new drugs and in understanding possible resistance mechanisms.

## Introduction

The cell wall is critical for the interaction of fungal cells with their environment. It provides a resilient framework that permits survival over a wide range of environmental conditions. It is also the point of contact between fungal cells and surfaces to which they may bind. As the determinant of fungal cell shape, it is modified during morphogenetic programs, including budding, mating, hypha production, sporulation, and host invasion by pathogens; genes specifying cell wall proteins and biogenesis enzymes are major targets of developmental regulatory pathways. As a distinguishing fungal structure, the cell wall is the target of natural antifungal metabolites and derivatives of growing therapeutic utility. Thus an understanding of the cell wall and its regulation is relevant to fungal ecology, development, and pathogenesis.

Our interest focuses on the cell wall of *Candida albicans,* the major invasive fungal pathogen of humans. The C. albicans cell wall is mainly composed of β-1,3-glucan and has significant content of β-1,6-glucan, chitin, and protein as well [1,2]. Molecular studies have begun to dissect C. albicans cell wall functions in adherence, nutrient acquisition, environmental adaptation, and morphogenetic programs. Such analyses have highlighted the role of cell wall functions in virulence [3,4]. The C. albicans cell wall is also of interest as the target of antifungal echinocandin drugs such as caspofungin. Caspofungin inhibits synthesis of β-1,3-glucan in both C. albicans and in the budding yeast Saccharomyces cerevisiae [5]. As a consequence, it provokes a broad transcriptional response in both organisms [6–8]. The response seems geared toward modification and repair of the cell wall, based primarily on the extensive study of gene function and regulatory relationships from *S. cerevisiae.*

A major determinant of caspofungin sensitivity in S. cerevisiae is the cell wall integrity (CWI) mitogen-activated protein kinase (MAPK) pathway [8–10]. This cascade receives numerous inputs from plasma membrane sensors and signaling molecules. These inputs converge upon G-protein ScRho1, which activates protein kinase ScPkc1. (We use the prefixes “Sc” and “Ca” to indicate S. cerevisiae and C. albicans gene products, respectively.) ScPkc1 in turn activates a MAPK cascade with both transcriptional and nontranscriptional outputs. The transcriptional output is mediated by two transcription factors, ScRlm1 and ScSwi4/6. ScRlm1 is a key activator of many cell wall protein genes and is required for resistance to numerous cell wall perturbing treatments and for activation of most known CWI pathway-responsive genes [11,12]. ScSwi4/6 is known primarily as an activator of G1 phase cell cycle-regulated genes, but it has been implicated in the CWI pathway gene expression response though biochemical and functional analyses [9].

The S. cerevisiae gene expression response to caspofungin has been probed through microarray analysis [6,8]. Agarwal et al. [6] reported significant up-regulation by caspofungin of ten ScRlm1-dependent genes—half of all known genes activated by ScRlm1 [11]—as well as Sc*RLM1* itself. Reinoso-Martin et al. [8] reported up-regulation by caspofungin of four ScRlm1-dependent genes and again detected up-regulation of Sc*RLM1.* These studies agree that ScRlm1-dependent transcription contributes to the overall response to caspofungin, and the fact that several ScRlm1-dependent genes specify cell wall proteins emphasizes the likelihood that this aspect of the response is functionally significant.

The S. cerevisiae model has been extended to C. albicans through both mutant and microarray analyses. Mutant analysis, driven by a candidate-gene approach, indicates that C. albicans CaPkc1 as well as the CWI MAPK homolog, CaMkc1, are required for cell wall damage responses [13–15]. Microarray studies show that caspofungin induces expression of several cell wall protein and cell wall maintenance genes [7], including two genes whose S. cerevisiae homologs are activated by ScRlm1. Thus there is the expectation that C. albicans responds to caspofungin through gene products homologous to the S. cerevisiae CWI pathway components, following mechanisms elucidated in *S. cerevisiae.*

In contrast to that expectation, we describe here a new zinc finger protein, CaCas5, which is required for expression of numerous caspofungin-responsive genes. Mutant analysis suggests that CaCas5 has a major functional role in this response and that the unique role of CaRlm1 is more limited. We also find unique features of the C. albicans caspofungin transcriptional response that parallel S. cerevisiae functional analysis more closely than gene expression analysis. Thus divergent aspects of gene expression responses may provide generally useful guides to direct mutant analysis.

## Results

### Identification of Transcription Factor Mutants Hypersensitive to Cell Wall Perturbation

In order to identify C. albicans regulators of the cell wall damage response, we screened among mutants homozygous for insertions in 83 putative transcription factor genes [16] for altered growth on medium containing caspofungin. Mutants with insertions in any of five genes were hypersensitive to caspofungin, including Ca*CAS1,* Ca*FGR15* (Ca*CAS2*), Ca*ADA2* (Ca*CAS3*), Ca*CAS4,* and Ca*CAS5.* (The gene name CAS stands for caspofungin sensitivity.) We did not identify resistant mutants.

**Figure 1:**
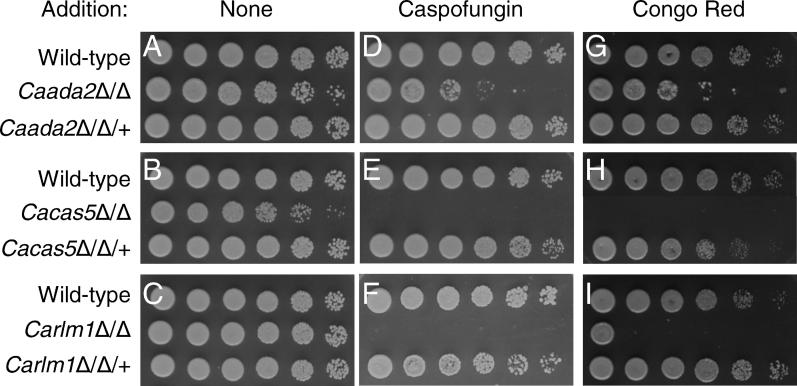
Growth of Wild-Type, Mutant, and Complemented Strains on Caspofungin Medium Serial dilutions of an overnight culture were spotted on YPD medium with no addition (A–C), 25 ng/ml caspofungin (D and E), 125 ng/ml caspofungin (F), or 50 μg/ml Congo red (G–I). Growth was visualized after 1–2 d at 30 °C. The wild-type reference strain (DAY185), mutant (Δ/Δ), and complemented (Δ/Δ/+) strains are shown. All strains were prototrophic; detailed genotypes are listed in Table S2.

We took three steps to verify that these genes influence the sensitivity of C. albicans to caspofungin. First, we tested multiple isolates of each mutant where possible. These results confirmed the findings from the initial screen. Second, we created deletion mutants for each gene. We observed that Ca*cas1*Δ/Δ and Ca*fgr15*Δ/Δ strains showed only marginal hypersensitivity to the drug. We were unable to create a Ca*cas4*Δ/Δ strain, nor were we able to create homozygous Ca*cas4*::*Tn7*/Ca*cas4*::*Tn7* insertion mutants with *Tn7* insertions at codons 836, 842, 1163, and 1828. The viable Ca*cas4*::*Tn7*/Ca*cas4*::*Tn7* mutant has an insertion near codon 2570, very close to the 3′ end of the open reading frame (codon 2831). Thus we believe that Ca*CAS4* is an essential gene, as is its homolog Sc*TAO3* in some S. cerevisiae strains, and that the viable insertion mutant has a partial defect in Ca*CAS4* function. However, viable Ca*ada2*Δ/Δ and Ca*cas5*Δ/Δ strains were constructed and showed significant hypersensitivity to caspofungin (Figure 1). The third verification step was to complement each hypersensitive deletion mutant by introduction of a wild-type copy of the corresponding gene. Complementation tests verified that Ca*ada2*Δ and Ca*cas5*Δ mutations cause caspofungin hypersensitivity (Figure 1A, 1B, 1D, and 1E). Therefore, Ca*ADA2* and Ca*CAS5* are required for normal growth of C. albicans in the presence of caspofungin, and the roles of Ca*CAS1,* Ca*CAS2,* and Ca*CAS4* may be minor or complex.

The transcription factor ScRlm1 plays a major role in the S. cerevisiae CWI pathway [9]. Thus we tested a C. albicans Ca*rlm1*Δ/Δ mutant and complemented derivative for sensitivity to caspofungin. We observed slight growth inhibition of the Ca*rlm1*Δ/Δ mutant on our typical caspofungin-containing medium (unpublished data) and a more severe growth defect at higher caspofungin concentrations (Figure 1C and 1F). Complementation with a cloned copy of Ca*RLM1* restored growth that was comparable to the wild-type reference strain (Figure 1C and 1F). Thus Ca*RLM1* is required for normal growth in the presence of caspofungin.

The mutants above may be hypersensitive only to caspofungin or may have a more global defect in cell wall structure or integrity. To distinguish between these explanations, we tested growth of the mutants on two additional cell wall perturbing compounds, Congo red and sodium dodecyl sulfate [17,18]. All three mutants were hypersensitive to Congo red (Figure 1G, 1H, and 1I), and the Ca*ada2*Δ/Δ and Ca*cas5*Δ/Δ mutants were hypersensitive to sodium dodecyl sulfate (unpublished data). We conclude that Ca*RLM1,* Ca*ADA2,* and Ca*CAS5* functions are not specific for interaction with caspofungin, but are required more generally for cell wall structure or integrity.

### Transcriptional Response to Caspofungin

Ca*RLM1,* Ca*ADA2,* and Ca*CAS5* specify putative transcription factors. Thus the mutant phenotypes arise as a consequence of altered gene expression. Because the mutant phenotypes are apparent in the presence of caspofungin, we focused on caspofungin-responsive gene expression.

**Figure 2:**
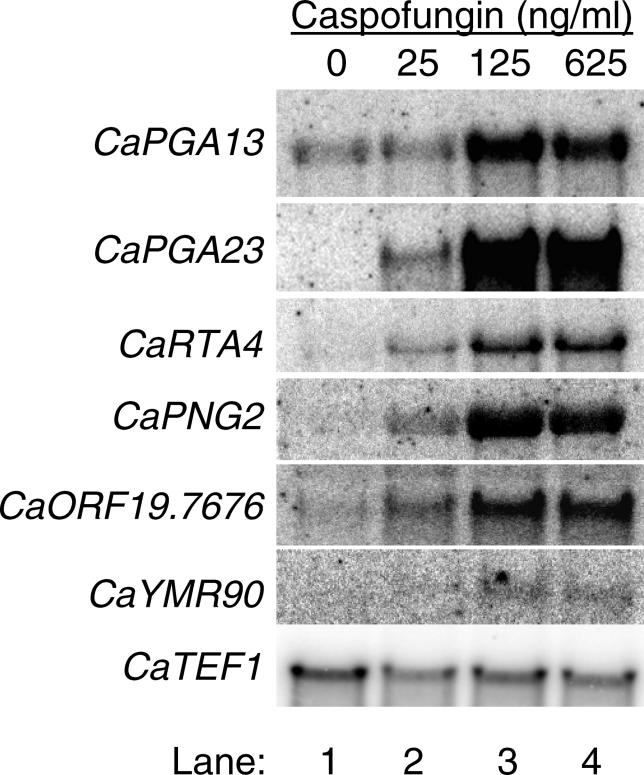
Expression of Caspofungin-Responsive Genes Wild-type reference strain DAY185 was grown in YPD medium at 30 °C to midexponential phase, and aliquots of the culture received caspofungin at the indicated final concentration. After 1 h of further incubation, RNA was prepared for Northern blot analysis and probed for the indicated transcripts. Ca*TEF1* was used as a loading control.

Pilot microarray studies identified several caspofungin-induced genes, for which we then optimized induction conditions. We examined expression of six genes after an hour of drug treatment and found that 25 ng/ml caspofungin led to partial induction, whereas 125 and 625 ng/ml led to similar levels of induction (Figure 2). This analysis indicated that a 1 h incubation in 125 ng/ml caspofungin would be suitable to elicit some gene expression responses. As this concentration inhibits growth of all three mutants, it seemed reasonable for comparison of wild-type and mutant strains as well.

**Figure 3:**
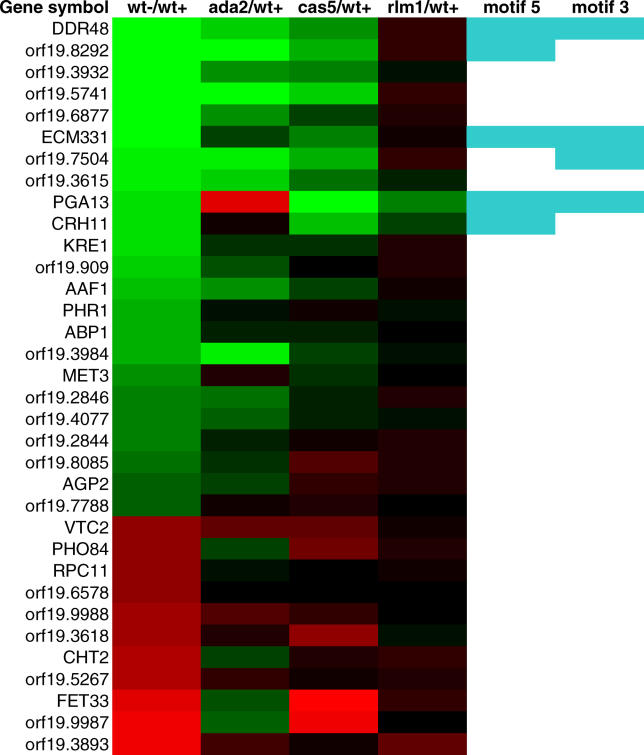
The Core Set of C. albicans Caspofungin-Responsive Genes The expression profiles of 34 caspofungin-responsive genes that (i) exhibited a 2-fold change (*p* < 0.05) in response to caspofungin treatment in our experiments (Dataset S1) and (ii) exhibited a corresponding increase or decrease in response to caspofungin in the report by Liu et al. [7]. “Gene symbol” refers to entries in CGD. The columns “wt−/wt+,” “*ada2*/wt+,” “*cas5*/wt+,” and “*rlm1*/wt+” summarize the response of each gene in microarray comparisons of the untreated wild-type strain, the treated Ca*ada2*Δ/Δ strain, the treated Ca*cas5*Δ/Δ strain, and the treated Ca*rlm1*Δ/Δ strain, each compared to the treated wild-type strain, respectively. Green color indicates a higher level of expression in the treated wild-type strain; red color indicates a lower level of expression in the treated wild-type strain. The degree of color saturation represents the magnitude of the expression ratio, which may be found in Dataset S1. The columns “motif 5” and “motif 3” refer to presence of the nucleotide sequences GTGGYSYKGKKG and WTGWTRWTGWTGWKSYKGW, respectively, within 999 bp of each gene's 5′ region; blue color indicates presence of the sequence. The motifs were identified through MEME analysis of CaCas5-dependent gene 5′ regions. Among noncore gene 5′ regions, motif 5 was also associated with orf19.7350, orf19.6595, orf19.753, and orf19.4771; motif 3 was also associated with orf19.13071, orf19.6595, orf19.753, orf19.7350, orf19.4771, and orf19.711.

In order to identify caspofungin-responsive genes on a large scale, we conducted microarray comparisons of a wild-type reference strain (DAY185) with or without caspofungin treatment for 1 h. Based upon statistical confidence (*p* < 0.05) and an expression change of at least 2-fold, we identified 216 caspofungin-responsive genes, including 170 up-regulated genes and 46 down-regulated genes (Dataset S1). Fifty-four of these caspofungin-responsive genes were also analyzed by Liu et al. [7] in a study employing different treatment conditions, a different microarray platform, and the related wild-type strain SC5314. Our assessments of the direction of expression changes correlate with the Liu study for 34 genes, including 23 up-regulated genes and 11 down-regulated genes (Figure 3). We refer to these 34 genes as the core set of caspofungin-responsive genes, indicating that their responses are sufficiently resilient to be detected under diverse conditions.

### Relationship between Caspofungin-Sensitive Mutants and Caspofungin-Responsive Genes

To determine whether the caspofungin-sensitive mutants were defective in regulation of caspofungin-responsive genes, we conducted microarray comparisons of each mutant and reference strain DAY185, both treated with caspofungin for 1 h (Figure 3; Dataset S1). Each mutant expressed several caspofungin-responsive genes at levels comparable to the reference strain (black rectangles in lanes of Figure 3), so that no mutant is completely defective in this gene expression response. This observation may indicate that the response to caspofungin integrates activities of several transcriptional regulatory pathways.

The Ca*rlm1*Δ/Δ mutant had a fairly mild gene expression alteration. It failed to fully express only four up-regulated genes (one of which was Ca*RLM1* itself) and overexpressed two additional up-regulated genes. Our dataset includes three genes up-regulated by caspofungin whose S. cerevisiae homologs are activated by ScRlm1: Ca*CRH11,* Ca*ECM331,* and Ca*DFG5.* Ca*ECM331* and Ca*DFG5* were expressed at similar levels in the wild-type reference strain and the Ca*rlm1*Δ/Δ mutant; Ca*CRH11* expression was reduced slightly in the mutant (Figure 3; Dataset S1). These observations suggest that a different transcription factor may have assumed the key functional role in CWI signaling in *C. albicans.*

The Ca*ada2*Δ/Δ mutant had the most severe gene expression alteration. It failed to fully express 59 up-regulated genes; it overexpressed nine up-regulated genes; it failed to fully repress five down-regulated genes; and it hyperrepressed five down-regulated genes. The mutant also had altered expression of 180 genes that were not significantly responsive to caspofungin (Dataset S1). CaAda2 homologs in other eukaryotes have broad roles in gene expression as transcriptional coactivators [19]. The fact that the Ca*ada2*Δ/Δ mutant has such a pleiotropic effect on gene expression fits well with the idea that C. albicans CaAda2 is a coactivator, functioning in diverse regulatory pathways.

**Figure 4:**
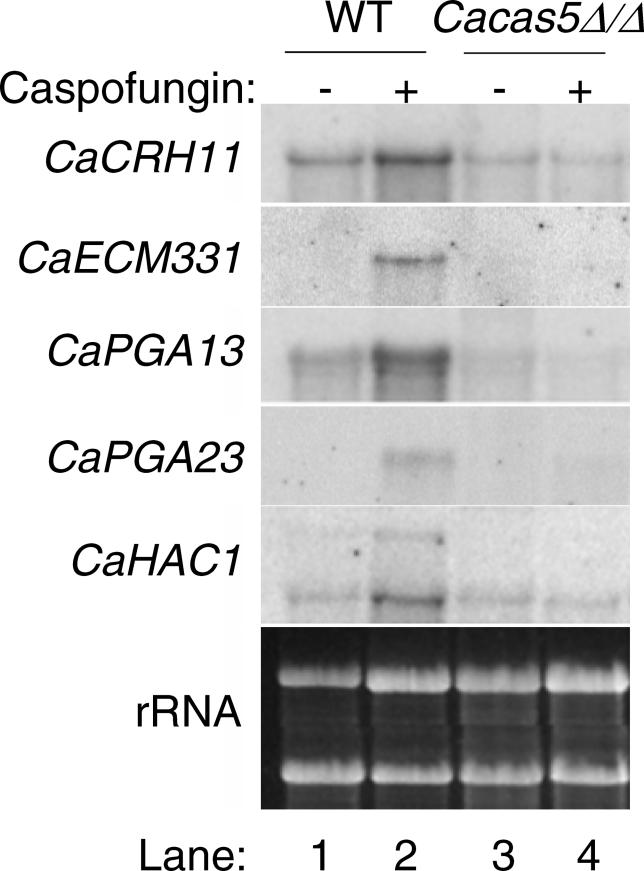
Dependence of Caspofungin-Responsive Genes on CaCas5 Reference strain DAY185 and prototrophic Ca*cas5*Δ/Δ mutant VIC1186 were grown and treated with 125 ng/ml caspofungin as described for Figure 2. RNA was prepared for Northern blot analysis and probed for the indicated transcripts. The rRNA bands visualized with ethidium bromide staining were used as a loading control.

The Ca*cas5*Δ/Δ mutant also had severe gene expression defect. It failed to fully express 37 up-regulated genes and failed to repress nine down-regulated genes (Figure 3; Dataset S1). Noteworthy was its defect in expression of Ca*CRH11* and Ca*ECM331,* two caspofungin-induced homologs of ScRlm1-dependent genes, and seven other core caspofungin-inducible genes (Figure 3). Northern analysis confirmed its expression defect for core genes Ca*CRH11,* Ca*ECM331,* and Ca*PGA13,* as well as Ca*PGA23* and Ca*HAC1* (Figure 4). Because the mutant had altered expression of only 15 genes that were not significantly responsive to caspofungin (Dataset S1), CaCas5 function seems to be substantially specific for cell wall damage-responsive gene expression.

## Discussion

### Caspofungin-Responsive Gene Function in C. albicans

Our current understanding of fungal cell wall damage responses comes largely from studies in *S. cerevisiae,* an essential point of comparison. We found 125 C. albicans caspofungin-responsive genes whose S. cerevisiae homologs were described in two studies of that organism's response to caspofungin [6,8]. Only five homologs of genes up-regulated in our study were up-regulated in both S. cerevisiae studies: Ca*ECM331*/Sc*PST1,* Ca*CRH11*/Sc*CRH1,* Ca*DFG5*/Sc*DFG5,* Ca*RLM1*/Sc*RLM1,* and Ca*GYP7*/Sc*GYP7.* The first three genes depend upon ScRlm1 for their expression in S. cerevisiae and encode inferred or proven GPI-linked cell surface proteins that have been implicated in cell wall biogenesis or repair [20–23]. Ca*RLM1/*Sc*RLM1* governs cell wall functions in both organisms, as discussed below. Up-regulation of cell wall functions is also reflected in the core C. albicans responses (*p* = 7.95 × 10^−11^ for 9/25 S. cerevisiae homologs; http://db.yeastgenome.org/cgi-bin/GO/goTermFinder) and in our overall dataset. Thus both conserved and overall C. albicans caspofungin-induced genes have a close functional relationship to cell wall biogenesis or repair, as noted previously for S. cerevisiae [6,8].

The conserved up-regulation of Ca*GYP7*/Sc*GYP7* may reflect the need for surface export functions during cell wall repair. Sc*GYP7* specifies the GTPase-activating protein for G-Protein ScYpt1, which promotes Golgi-to-vacuole transport [24]. Up-regulation of CaGyp7 may thus inhibit transport to the vacuole, perhaps favoring transport of material from the Golgi to the cell surface. Two prior findings with S. cerevisiae suggest that cell surface transport is limiting for cell wall repair: overexpression of Golgi-to-cell surface transport stimulator Sc*SBE2* confers resistance to caspofungin [25], and defects in secretion cause hypersensitivity to caspofungin [10]. Among our overall set of caspofungin-induced C. albicans genes, there is a clear representation of cytoplasmic vesicle or organelle lumen functions (*p* = 2.47 × 10^−5^ for 4/108 S. cerevisiae homologs or 0.01588 for 6/144 C. albicans genes; http://www.candidagenome.org/cgi-bin/GO/goTermFinder). Noteworthy also is up-regulation of Ca*HAC1* (Figure 4); its homolog Sc*HAC1* specifies the transcriptional activator of the unfolded protein response, an endoplasmic reticulum stress-response pathway [26,27]. However, secretion functions are not represented among caspofungin-induced S. cerevisiae genes [6,8]; instead, they are down-regulated in one set of experiments (*p* = 0.00791 for 3/133 genes [8]). The symmetry between the genes induced in C. albicans and those required in S. cerevisiae in the presence of caspofungin illustrates that divergent gene expression responses may highlight conserved functional relationships.

There is only one homolog pair whose down-regulation after caspofungin treatment is conserved: Ca*HAS1*/Sc*HAS1.* ScHas1 is a putative helicase required for rRNA processing and ribosome biogenesis, and its expression is down-regulated in response to numerous environmental stresses [28]. Down-regulation of ribosome biogenesis functions is also reflected in our overall dataset (*p* = 0.00576 for 6/42 C. albicans genes). This response to caspofungin may reflect a coupling between cell wall biogenesis and overall cell growth.

### Role of CaRlm1 in CWI

Our analysis of Ca*RLM1* here was motivated by a candidate-gene approach, based on the central role of ScRlm1 in the S. cerevisiae CWI pathway. This role is emphasized by the fact that Ca*RLM1* expression is induced by caspofungin in both C. albicans and *S. cerevisiae.* The drug hypersensitivity of the C. albicans Ca*rlm1*Δ/Δ mutant reported here implicates CaRlm1 in the C. albicans cell wall damage response, thus arguing that its biological function is conserved.

Although conservation of CaRlm1 biological function is expected, the fact that we found so few CaRlm1-dependent genes seems surprising. The major caspofungin-inducible Rlm1-dependent gene we detected was Ca*PGA13* (orf19.6420), a gene in the core set that specifies a predicted GPI-linked cell wall protein without close homologs. It is possible that CaPga13 is required for C. albicans cell wall repair. We also detected partial CaRlm1-dependence of Ca*CRH11,* a result that has been confirmed by Northern analysis (unpublished results). Thus there may be only two cell wall-related targets of CaRlm1. A second possibility is that CaRlm1-dependent genes are induced only at earlier or later time points than we have examined here.

### Role of CaAda2 in CWI

CaAda2 homologs are subunits of transcriptional coactivator complexes that are recruited by site-specific DNA binding proteins such as ScGcn4 and ScGal4 to activate transcription [29]. Our microarray results are consistent with the hypothesis that C. albicans CaAda2 functions in conjunction with many different regulators, because CaAda2-responsive genes are more numerous than CaRlm1- or CaCas5-responsive genes and extend well beyond the set of caspofungin-responsive genes.

There is a substantial effect of the Ca*ada2*Δ/Δ mutation on caspofungin-responsive genes. These results may be explained by either indirect or direct roles of CaAda2 in this response. One hypothetical indirect role is that CaAda2 may be required for expression of genes that are in turn required for caspofungin-induced gene expression. The fact that CaAda2-responsive genes are so numerous makes this explanation difficult to test. A hypothetical direct role of CaAda2 is that it may be recruited to caspofungin-induced gene regulatory regions through interaction with a site-specific DNA binding protein, where it participates directly in their transcriptional activation. Given that 25 of the 30 most highly CaCas5-dependent genes are also CaAda2-dependent, this explanation predicts that CaCas5 may activate transcription through recruitment of CaAda2. One prediction of this hypothesis is that a double Ca*ada2*Δ/Δ Ca*cas5*Δ/Δ mutant should have the same phenotype as one or the other single mutant. We have been unable to construct the double mutant with methods that are routinely successful in this lab, and it is possible that the two mutations are synthetically lethal. Thus a critical test of this hypothesis will require other strategies.

### Role of CaCas5 in CWI

CaCas5 has a major role in the C. albicans cell wall damage response. Among the mutants we have screened, it is the most sensitive to both caspofungin and Congo red. It is required for full expression of 15 of the 30 genes most highly induced by caspofungin, and nine genes in the C. albicans core set. Noteworthy among these are Ca*ECM331* and Ca*CRH11,* whose S. cerevisiae homologs are ScRlm1-dependent. In general, the major class of caspofungin–induced CaCas5-dependent genes include cell wall functions (*p* = 1.75 × 10^−5^ for S. cerevisiae homologs; 5/22 genes). Thus C. albicans CaCas5 may be the functional equivalent of S. cerevisiae ScRlm1.

The closest S. cerevisiae homolog of CaCas5 is ScMig2, a repressor of glucose-repressible genes. However, the C. albicans orf19.5326 product, rather than CaCas5, appears to be the ScMig2 ortholog. There are now several examples of novel transcriptional regulators in C. albicans with key biological functions, as well as conserved transcriptional regulators with divergent functions in C. albicans and S. cerevisiae [16,30–35]. These two considerations—functionally divergent homologs and unique key regulators—underscore the importance of analyzing C. albicans gene function de novo, rather than relying solely upon inferences from *S. cerevisiae.*

Similarity of CaCas5 and ScMig2 is confined to their two C-terminal zinc fingers, but the degree of similarity (51% identity) does not provide confidence that Cas5 binds to the Mig2 consensus binding site, ATAAAAATGCGGGGAA [36]. We have used MEME analysis (http://meme.sdsc.edu/meme/website/intro.html [37]) of CaCas5-responsive gene 5′ regions to look for motifs that may represent a CaCas5-responsive site or CaCas5 binding site. We focused on 999 base pairs of 5′ sequence from the 13 top CaCas5-dependent genes. One motif, WTGWTRWTGWTGWKSYKGW (motif 3), was found in ten of the 13 promoter regions, including four core genes (Figure 3; Protocol S1; Figure S1). The site seems credible because its length is similar to that of the ScMig2 binding site (19 and 17 bp, respectively) and because the best matches are found among the more highly CaCas5-dependent genes. A second shorter motif, GTGGYSYKGKKG (motif 5), was found among nine promoter regions, including five core genes (Figure 3). Our working hypothesis is that one of these sequences may be a CaCas5 binding site.

Our findings provide some insight into the relationships among CaCas5, its target genes, and the role of CaCas5 in the cell wall damage response. They also raise the question of whether and how CaCas5 responds to cell wall damage. The simplest possibility is that CaCas5 is a target of the protein kinase C–MAPK pathway. The C. albicans MAPK of this pathway, CaMkc1, clearly functions in cell wall damage responses [13,14]. Our preliminary results indicate that CaMkc1, the MAPKKK homolog CaBck1, and the MAPKK homolog CaMkk2 are required for growth in the presence of caspofungin, in keeping with this model. A second possibility is that CaCas5 is the target of a different pathway that responds to cell wall damage. There are several good candidates—the calcineurin or high-osmolarity pathways, for example—based on studies in S. cerevisiae [9]. It is also possible that CaCas5 functions in a novel C. albicans pathway. We anticipate that these many possibilities may be addressed by screening C. albicans mutants defective in other kinds of gene products, such as protein kinases or membrane proteins, for defects that might be expected from altered CaCas5 activity.

## Materials and Methods

### Media and chemicals.

C. albicans strains were routinely passaged in YPD plus uridine (2% dextrose, 2% Bacto Peptone, 1% yeast extract, and 80 mgl/l uridine) at 30 °C. Following transformation, selection was accomplished on synthetic medium (2% dextrose, 6.7% yeast nitrogen base (YNB) plus ammonium sulfate, and the necessary auxotrophic supplements). Caspofungin was a generous gift from Merck.

### Yeast strains and DNA manipulations.

All strains used in this study were derived from strain BWP17 (genotype: *ura3Δ*::λ*imm434*/*ura3Δ*::λ*imm434 his1*::*hisG*/*his1*::*hisG arg4*::*hisg*/*arg4*::*hisG* [38]) through standard transformation methods [38]. Details of these manipulations and complete genotypes are given in Protocol S1, Table S1, and Table S2. The transcription factor mutant collection has been described [16].

### Caspofungin susceptibility tests.

Single colonies were inoculated into 3 ml of YPD and grown overnight at 30 °C. These overnight cultures were then diluted in YPD to an OD_600_ of 3.0, which was used as a starting point for 5-fold serial dilutions in YPD. 3 μl of each serial dilution, beginning with the OD_600_ = 3.0 dilution, was spotted onto the appropriate plates, allowed to dry, and incubated at 30 °C. The plates were photographed after 1 or 2 d of growth.

### Gene expression measurements and microarray data analysis.

Single colonies were inoculated into 3 ml of YPD and grown overnight at 30 °C. Each overnight culture was used to inoculate 200 ml of YPD to an OD_600_ of 0.1, which was then incubated at 30 °C with shaking for 2–3 doublings. At this point the cultures were divided into two 100-ml cultures. To one of the cultures, caspofungin (diluted in dH_2_0) was added to a final concentration of 125 ng/ml. To the other culture an equal volume of dH_2_0 was added. The cultures were then incubated with shaking at 30 °C for 1 h, at which point they were harvested by vacuum filtration and stored at −80 °C. RNA isolation, microarray analysis, and Northern analysis were performed as previously described [16]. Microarray slides were scanned using ScanArray Express microarray scanner (PerkinElmer, Wellesley, California, United States), and the signal intensities were extracted with GenePix Pro 4.1 software (Axon Instruments, Union City, California, United States). Raw signal intensities were corrected for dye labeling effects within and between all slides using the normalize.loess R-function [39] implemented in an *affy* microarray analysis package [40]. The resulting data were imported into the Spotfire DecisionSite for Functional Genomics software suite (Spotfire, Somerville, Massachusetts, United States) and filtered according to the GenePix quality scores above 0 and signal-to-noise ratio above 2. The *p*-values for differentially expressed genes between the compared strains were subsequently calculated and further adjusted for type I error with Bonferroni's transformation using BioConductor multtest R-package [41] (see http://cran.r-project.org/doc/packages/multtest.pdf). The results of this analysis with adjusted *p*-values below 0.05 and absolute fold changes above 2 are listed in Dataset S1. All told, we compared three hybridizations with untreated wild-type cell samples and 12 hybridizations with drug-treated wild-type cell samples to identify caspofungin-responsive genes. We compared three hybridizations of drug-treated mutant cells to drug-treated wild-type cells to identify dependent genes for each mutant. RNA for each sample came from an independent culture.

## Supporting Information

Dataset S1 Microarray Analysis of Caspofungin-Responsive Gene Expression These data compare the wild-type plus and minus treatment, the wild-type to each mutant, and our data to related published S. cerevisiae and C. albicans data.
(6.1 MB XLS)

Figure S1 MEME Analysis Results This figure shows the results of 5′ region MEME analysis.
(315 KB DOC)

Protocol S1 Supplementary Methods (58 KB DOC)

Table S1 Oligonucleotide Sequences (48 KB DOC)

Table S2 Complete Genotypes of C. albicans Strains (37 KB DOC)

### Accession Numbers

Data for the following genes and mutant alleles (with systematic names in parentheses) are deposited in the *Candida* Genome Database (http://www.candidagenome.org): Ca*CAS1* (orf19.1135), Ca*FGR15* or Ca*CAS2* (orf19.2054), Ca*ADA2* or Ca*CAS3* (orf19.2331), Ca*CAS4* (orf19.9261), Ca*CAS5* (orf19.4670), Ca*RLM1* (orf19.4662), and Ca*PGA13* (orf19.6420).

## Abbreviations

CWI: cell wall integrity
MAPK: mitogen-activated protein kinase
