# Supplementary material for: Control of the C. albicans Cell Wall Damage Response by Transcriptional Regulator Cas5
Source: PLoS Pathog. 2006 Mar 17;2(3):e21. doi: 10.1371/journal.ppat.0020021 (PMC1401495; doi:10.1371/journal.ppat.0020021)
Supplement: Supplementary file 1 [file ppat.0020021.sd002.doc]

Plasmid constructions

The primers used in this study are listed in Table S1.

The *CaADA2* reconstruction plasmid (pDDB78-*ADA2*) was generated as follows. PCR, using primers ADA2-5comp and ADA2-3comp, was used to generate a 3257 bp fragment (which includes the complete ORF, 1628 bp of promoter sequence, and 291 bp of 3’UTR. This fragment was cloned into pGEMT-Ez and then transferred to NotI-linearized pDDB78 (1) using *in vivo* recombination to generate plasmid pDDB78-*ADA2.*

The *CaCAS5* reconstruction plasmid (pDDB78-*CAS5*) was generated as follows. PCR, using primers, CAS5-5comp and CAS5-3comp, was used to generate a 3905 bp fragment (which includes the complete ORF, 1082 bp of promoter sequence and 357 bp of 3’ UTR). This fragment was cloned into pGEMT-Ez and then transferred to NotI-linearized pDDB78 (1) using *in vivo* recombination to generate plasmid pDDB78-*CAS5*.

The *CaRLM1* reconstruction plasmid (pDDB78-*RLM1*) was generated as follows. PCR, using primers, RLM1-5comp and RLM1-3comp, was used to generate a 3223 bp fragment (which includes the complete ORF, 989bp of promoter sequence and 398 bp of 3’ UTR). This fragment was cloned into pGEMT-Ez and then transferred to NotI-linearized pDDB78 using *in vivo* recombination to generate plasmid pDDB78-*RLM1*.

Yeast strains and DNA manipulations

All strains used in this study were derived from strain BWP17 (genotype: *ura3∆::λimm434/ura3∆::λimm434 his1::hisG/his1::hisG arg4::hisg/arg4::hisG*; (2)) through standard transformation methods (2). Strains constructed for this study are listed in Table S2.

The *Caada2∆/Caada2∆* mutant, VIC1057, was generated as follows. Strain BWP17 was subjected to consecutive rounds of transformation with PCR products *ada2::ARG4* and *ada2::URA3* that were generated using primers ADA2-5DR and ADA2-3DR, following the method of Wilson *et al* (2). VIC1197 was generated by transforming VIC1057 to histidine prototrophy with NruI-linearized pDDB78-*ADA2* to target integration to the *HIS1* locus. VIC1057 was also transformed with NruI-digested pDDB78 to create VIC1145, VIC1151 and VIC1194.

The *Cacas5∆/Cacas5∆* mutant, VIC1075, was generated as follows. Strain BWP17 was subjected to consecutive rounds of transformation with PCR products *cas5::ARG4* and *cas5::URA3* that were generated using primers CAS5-5DR and CAS5-3DR, following the method of Wilson *et al* (2). VIC1190 was generated by transforming VIC1075 to histidine prototrophy with NruI-linearized pDDB78-*CAS5* to target integration to the *HIS1* locus. VIC1075 was also transformed with NruI-deigested pDDB78 to create VIC1147, VIC1152 and VIC1186.

The *Carlm1∆/Carlm1∆* mutant, VIC1090, was generated as follows. Strain BWP17 was subjected to consecutive rounds of transformation with PCR products *rlm1::ARG4* and *rlm1::URA3* that were generated using primers RLM1-5DR and RLM1-3DR, following the method of Wilson *et al* (2). VIC1209 was generated by transforming VIC1090 to histidine prototrophy with NruI-linearized pDDB78-*RLM1* to target integration to the *HIS1* locus. VIC1090 was also transformed with NruI-digested pDDB78 to create VIC1149, VIC1153 and VIC1206.

MEME analysis

To identify possible conserved motifs among CaCas5-responsive genes, we searched among 999 bp of 5’ flanking regions for the most highly CaCas5-dependent genes, including orf19.6420, orf19.753, *CaALS1*, orf19.13071, orf19.6595, orf19.2706, orf19.711, orf19.7504, orf19.8292, orf19.4082, orf19.7350, orf19.4771, and orf19.4255, using an online tool (<http://meme.sdsc.edu/meme/website/intro.html>). Details of our parameters will be provided upon request, as will our complete results. The motif 3 and 5 occurrences and probability matrices are shown in Figure S1.

References

1.	Spreghini, E., Davis, D. A., Subaran, R., Kim, M. & Mitchell, A. P. (2003) *Eukaryot Cell* **2,** 746-55.

2.	Wilson, R. B., Davis, D. & Mitchell, A. P. (1999) *J Bacteriol* **181,** 1868-74.

3.	Davis, D., Edwards, J. E., Jr., Mitchell, A. P. & Ibrahim, A. S. (2000) *Infect Immun* **68,** 5953-9.
