# Supplementary material for: Control of the C. albicans Cell Wall Damage Response by Transcriptional Regulator Cas5
Source: PLoS Pathog. 2006 Mar 17;2(3):e21. doi: 10.1371/journal.ppat.0020021 (PMC1401495; doi:10.1371/journal.ppat.0020021)
Supplement: Supplementary file 2 [file ppat.0020021.sg001.doc]

Figure S1. Probability matrices for motifs 3 and 5.
