# Supplementary material for: Control of the C. albicans Cell Wall Damage Response by Transcriptional Regulator Cas5
Source: PLoS Pathog. 2006 Mar 17;2(3):e21. doi: 10.1371/journal.ppat.0020021 (PMC1401495; doi:10.1371/journal.ppat.0020021)
Supplement: Supplementary file 3 [file ppat.0020021.st001.doc]

Table S1. Synthetic Oligonucleotides

| Oligo Name | Sequence (5’-3’) |
| --- | --- |
| CAS3-5DR | TTCTCCAATACTCCAAATAACTGCAAAAAAAAAAGATAAGATACACAAATAAGTAAACAAGTCTCTACTCCATACTAGGAAAAAAAATATTTGTTTCCCAGTCACGACGTT |
| CAS3-3DR | TAAATCTTATTAAAGTCAAATTGGCAGAAATAATTGAACGTGGTATTTAAATCCAATTATATAAATCTATGCAAAACAAAAAAAAATGAAAAGGTGGAATTGTGAGCGGATA |
| CAS5-5DR | ATCAATTTTTGCTATTTTATTTCTATTCTAATTTATTTACTTTGCTTTTCATCCCACCCCTTTGTTGGTAAATATAGACTTTAACATATACT TTTCCCAGTCACGACGTT |
| CAS5-3DR | TTTTACCACAATTTGGCATTTGACATTCAAATGGTTTTTCTGAGGAATGACTTTTAAGATGACGTTTAACATGTTCTGGTCTTTGAAACCTGTGGAATTGTGAGCGGATA |
| RLM1-5DR | GTGCCTTCCCACCACCCCTCTAACGTCATCTACTTTTTTTTTTATAATTATTAATATTTCAAAACAACAATATTATCTTGTCACAACCATTGTTTCCCAGTCACGACGTT |
| RLM1-3DR | GAAATCTTTTCGTTTAATTGTAGTCAACAGCACAATCCAATCAAAAACAAGGTGACTCTTTATGCTTGTTCTTTCTTTCTATTCACTTGTGGTGGAATTGTGAGCGGATA |
| CAS3-5det | GAATCAACCATATCTTCCCCCATTCC |
| CAS3-3det | GCTGATGATTTGATACAACAAGAG |
| CAS5-5det | GCGGGTTATCTAGAATTAGACTGG |
| CAS5-3det | TTCTTGTCCTTTCACAAGTCCATGAATC |
| RLM1-5det | CACATGGTGATACCAAAACTCTT |
| RLM1-3det | GGGCTATAGCCCTAAGTCACGCAC |
| ADA2-5comp | GATCGTAATGAAAGCTCAAACAATCC |
| ADA2-3comp | CAACGGAATTATCATTTTCAGTTTGGA |
| CAS5-5comp | AAAAGTGGGCTGAAATTGTAGTGCTC |
| CAS5-3comp | CGCTGGTTTAAATGACAAAATGTTGA |
| RLM1-5comp | AGACCTTGCTACCCATTTGT |
| RLM1-3comp | GCAAATGAAACCAAAGAGTTC |
| CRH11u | GCTGCTAATGCTAATGCTGAC |
| CRH11d | CGGCTTTGATGGATTCCC |
| ECM331u | AGTCGCTGCCTTATTGACATC |
| ECM331d | GAAATGGTGACAGCACCTTTG |
| PGA13u | TTGCTGCTACCACTGTTTTGG |
| PGA13d | TTGAGTAGTATGGCTGCTTGG |
| RTA4u | TTGCTGCCACTTATGTTCCAG |
| RTA4d | TTGGCATCATCCCCATTC |
| IPF351u | TCTCATGGCAAGATTGGTCA |
| IPF351d | CCCCATTAGCAATGTTGTAGC |
| IPF19801u | TGTCATCAACAATTCCCAGTG |
| IPF19801d | TCGTCATGATGGTTTTTTTCA |
| IPF4959u | ATCCCCAGAAATCACCTCCC |
| IPF4959d | TAGGCCTTGATGGCGTCTTTA |
| PGA23u | ACTTGCTGACGCTGCTTCTG |
| PGA23d | GGCAACAGCATTCATTCCG |
| TEF1-5 | ATAGTCATAATCAATCATGGGT |
| TEF1-3 | CTTACATAATATTCAACTAGC |
