# Supplementary material for: Control of the C. albicans Cell Wall Damage Response by Transcriptional Regulator Cas5
Source: PLoS Pathog. 2006 Mar 17;2(3):e21. doi: 10.1371/journal.ppat.0020021 (PMC1401495; doi:10.1371/journal.ppat.0020021)
Supplement: Supplementary file 4 [file ppat.0020021.st002.doc]

Table S2. *Candida albicans* strains used in this study.

| Strain | Relevant Genotype^1^ | Refererence |
| --- | --- | --- |
| DAY185 | *pARG4::URA3::arg4::hisG/arg4::hisG pHIS1::his1::his1/his1::hisG* | Davis, D., Edwards, J. E., Jr., Mitchell, A. P. & Ibrahim, A. S. (2000) *Infect Immun* **68,** 5953-9. |
| VIC1057 | *ada2∆::ARG4/ada2∆::URA3 his1::hisG/his1::hisG* | This Study |
| VIC1075 | *cas5∆::ARG4/cas5∆::URA3 his1::hisG/his1::hisG* | This Study |
| VIC1090 | *rlm1∆::ARG4/rlm1∆::URA3 his1::hisG/his1::hisG* | This Study |
| VIC1145 | *ada2∆::ARG4/ada2∆::URA3 pHIS1::his1::hisG/his1::hisG* | This Study |
| VIC1147 | *cas5∆::ARG4/cas5∆::URA3 pHIS1::his1::hisG/his1::hisG* | This Study |
| VIC1149 | *rlm1∆::ARG4/rlm1∆::URA3 pHIS1::his1::hisG/his1::hisG* | This Study |
| VIC1151 | *ada2∆::ARG4/ada2∆::URA3 pHIS1::his1::hisG/his1::hisG* | This Study |
| VIC1152 | *cas5∆::ARG4/cas5∆::URA3 pHIS1::his1::hisG/his1::hisG* | This Study |
| VIC1153 | *rlm1∆::ARG4/rlm1∆::URA3 pHIS1::his1::hisG/his1::hisG* | This Study |
| VIC1186 | *cas5∆::ARG4/cas5∆::URA3 pHIS1::his1::hisG/his1::hisG* | This Study |
| VIC1190 | *cas5∆::ARG4/cas5∆::URA3 pCAS5::HIS1::his1::hisG/his1::hisG* | This Study |
| VIC1194 | *ada2∆::ARG4/ada2∆::URA3 pHIS1::his1::hisG/his1::hisG* | This Study |
| VIC1197 | *ada2∆::ARG4/ada2∆::URA3 pADA2::HIS1::his1::hisG/his1::hisG* | This Study |
| VIC1206 | *rlm1∆::ARG4/rlm1∆::URA3 pHIS1::his1::hisG/his1::hisG* | This Study |
| VIC1209 | *rlm1∆::ARG4/rlm1∆::URA3 pRLM1::HIS1::his1::hisG/his1::hisG* | This Study |

Footnotes:

1. All C. albicans strains are derived from strain BWP17 and have the genotype *ura3∆::λimm434/ura3∆:: λimm434 arg4::hisG/arg4::hisG his1::hisG/his1::hisG* except as indicated.
